# Clinical significance and pathogenesis of GBP5 in infectious mononucleosis associated liver injury

**DOI:** 10.1186/s13052-025-01907-x

**Published:** 2025-03-12

**Authors:** Yan Li, Jiamei Tang, Yulan Ma, Yujuan Yan, Fangfang Cheng, Kun Wang

**Affiliations:** 1 Department of Infectious Diseases, Children’s Hospital of Soochow University, Suzhou, 215000 China; 2 Department of Infectious Diseases, Children’s Hospital, Zhejiang University School of Medicine, National Clinical Research Center for Child Health, Hangzhou, 310000 China; 3 Department of Infectious Diseases, Affiliated Infectious Diseases Hospital of Soochow University, Suzhou, 215000 China

**Keywords:** GBP5, infectious mononucleosis, Liver injury, NLRP3 inflammasome

## Abstract

**Background:**

Infectious mononucleosis (IM) is a common disease in children; however, liver injury is its most common complication. However, the pathogenesis of IM complicated with liver injury is ambiguous. Thus, this study aimed to explore the potential mechanism of IM-associated liver injury.

**Methods:**

This study was conducted at the Children’s Hospital of Soochow University by collecting peripheral blood of 70 hospitalized children with IM. These patients were categorized into the liver injury (LIG, *n* = 35) and the non-liver injury groups (NLIG, *n* = 35), respectively. Subsequently, PBMCs and plasma were separated and obtained. PBMCs transcriptome sequencing was performed in two groups (5 cases in each group), and significantly differentially expressed genes (DEGs) were screened. Additionally, GO function enrichment, KEGG enrichment and GSEA analyses were performed. RT-PCR helped to detect the relative GBP5, NLRP3 and caspase-1 expressions in two groups (30 cases in each group) while the two groups’ caspase-1, IL-1β and IL-18 in plasma levels were measured by ELISA. Thus, clinical and laboratory datas of 60 hospitalized children with IM were evaluated.

**Results:**

Transcriptome sequencing results showed that 171 DEGs were screened in the NLIG group, compared with the LIG. Among them, 154 DEGs were up-regulated, and 17 were down-regulated, respectively. KEGG and GSEA analyses showed that IM-associated liver injury is correlated with a NOD-like receptor signaling pathway. Statistically significant differences were observed in the white blood cell and lymphocyte counts, CD3^+^CD4^+^ T cells, CD3^+^CD8^+^T cells, alanine aminotransferase (ALT), aspartate transaminase (AST), and lactate dehydrogenase (LDH) of the two groups (*p* < 0.05). Compared with NLIG, GBP5, NLRP3 and caspase-1 expressions in PBMCs, as well as the caspase-1, IL-1β and IL-18 in plasma levels, were significantly higher in LIG (*p* < 0.001). A correlation analysis revealed a positive correlation of GBP5 with LDH, ALT, AST, CD3^+^CD8^+^T cells and NLRP3 (*p* < 0.05).

**Conclusions:**

Our findings demonstrate that GBP5 contributes to liver injury in IM children through the NLRP3-dependent pathway.

## Background

A linear double-stranded DNA virus, Epstein-Barr virus (EBV) belongs to the herpesviridae family’s γ subfamily, herpesvirus type IV. It is a specific human lymphotropic herpesvirus that has latent and activation features. Although 90% of the population has the virus, most of them are asymptomatic [1]. EBV infection causes several diseases, including infectious mononucleosis (IM), chronic active EBV infection, and hemophagocytic syndrome. It is also associated with malignant and autoimmune diseases, like nasopharyngeal carcinoma, lymphoma, post-transplant lymphoproliferative disease, and gastric as well as breast cancers, respectively [2]. Moreover, some reports have shown that the EBV may induce diseases such as acute pancreatitis and encephalitis [3, 4].

IM is the most common clinical syndrome caused by primary EBV infection. The incidence of IM in Chinese children is high, with a peak incidence between four and six years [5]. Clinically, it manifests as fever, isthmitis, cervical lymphadenopathy, hepatomegaly, splenomegaly, and eyelid edema [6]. Additionally, EBV causes multi-organ functional damage, of which liver injury is the most common manifestation [7]. Moreover, 60-70% of IM pediatric patients exhibit liver injury, with the main clinical symptoms as elevated liver transaminase levels. Some pediatric patients may acquire acute cholestatic hepatitis due to abnormal bilirubin metabolism. However, few children may progress to acute liver failure, which indicates a poor prognosis [8]. At present, the pathogenesis of IM complicated with liver injury is ambiguous.

Human guanylate-binding protein 5 (GBP5) is a member of the dynamin superfamily of GTPases. Having a molecular weight of approximately 66 kDa, it has a highly homologous and conserved GTP binding or hydrolytic domain [9]. GBP5 helps in important cellular processes, including signal transduction, exocytosis, translation, and vesicle trafficking [10]. It is also an important regulator of inflammation and a cellular autoimmune component [11, 12]. It plays an important role in maintaining the host’s resistance to infection by pathogens, like viruses, bacteria, parasites, etc [13–15]. Additionally, GBP5 is an essential mediator of liver inflammation [16]. GBP5 is abnormally overexpressed in the liver of patients with cirrhosis, HBV infection, and HBV-related hepatocellular carcinoma, which is closely related to liver injury [17]. However, the GBP5 expression in IM-associated liver injury is not yet clear. Hence, this study aimed to examine the relationship between the GBP5 level and liver injury in IM pediatric patients as well as explore its potential mechanism.

## Materials and methods

### Study patients and design

We included 70 cases of hospitalized IM children from Children’s Hospital of Soochow University from January 2024 to June 2024. The criteria of IM were [18]:(1) the presence of at least three clinical manifestations: fever, isthmitis, cervical lymphadenopathy, hepatomegaly, splenomegaly, and eyelid edema; (2) the serological antibody test indicating primary EBV infection: presence of immunoglobulin (Ig)M and IgG to EBV viral capsid antigen (VCA-IgM and VCA-IgG)-positive, respectively, with absence of IgG to EB nuclear antigen (EBNA) or only VCA-IgG positive (low affinity). All patients were divided into two groups based on the presence of liver injury: the liver injury group (LIG) and the non-liver injury group (NLIG). The criteria for the LIG included a liver enzyme level increase of at least two times the upper limit of normal. Children with conditions such as nonalcoholic fatty liver disease, autoimmune hepatitis, biliary atresia or **inherited metabolic diseases(like Wilson disease**,** alpha-1 antitrypsin deficiency**,** hereditary hemochromatosis)** were excluded. Additionally, children who had used hepatotoxic drugs prior to admission or had infections like human immunodeficiency virus, hepatotropic viruses, or cytomegalovirus were also excluded. Routine blood tests and assays for detecting EBV-specific antibodies, alanine aminotransferase (ALT), aspartate transaminase (AST), total bilirubin (TBil), lactate dehydrogenase (LDH), lymphocyte subsets and immunoglobulin (Ig) were performed within 24 h of admission. The Ethics Committee of the Children’s Hospital of Soochow University, China gave ethical approval for this study (ethics approval number: 2023CS222). All the participants provided written informed consent.

### Extraction of human peripheral blood mononuclear cells

The centrifuge tube was first filled with human peripheral blood mononuclear cells (PBMCs) separation solution (2 mL) and then the IM patients’ venous blood (2 mL). The centrifuge tube was separated into four layers following a 650 g centrifugation for 30 min. The plasma layer was absorbed and transferred to the new centrifuge tube, followed by the transfer of the second layer in the same way. After adding the cleaning solution (10 mL), the contents were centrifuged at 250 g for 10 min, and the supernatant was discarded. After washing twice, as described above, the PBMCs were obtained after supernatant removal.

### Transcriptome sequencing

The PBMCs of ten IM children were collected and divided into NLIG and LIG based on the presence of liver injury. No significant difference was observed in sex and age in each group. After the total RNA extraction by TRIzol, quality inspection and transcriptome sequencing were completed. The two groups’ differential gene expression analysis was conducted using the DESeg R package. DESeg R package genes with an adjusted value < 0.05 were designated as the differentially expressed genes (DEGs). Subsequently, DEGs and functional enrichment analyses were completed.

### Data collection

The demographic, clinical, and laboratory characteristics were collected. Demographic and clinical characteristics included age, gender, presence and duration of fever as well as hospital stays. The laboratory values included white blood cells (WBC), neutrophils (N), lymphocytes (L), monocytes (M), immunoglobulins (Ig), lymphocyte subsets, plasma EBV-DNA, whole blood EBV-DNA and liver function tests.

### Quantitative real-time PCR

PBMCs were resuspended in TRIzol (0.5 mL, Thermo Fisher Scientific) to extract total RNA and subsequently reverse transcribed to synthesize cDNA. The SYBR Green PCR Master Mix system (Jiangsu Cowin Biotech Co., Ltd.) helped to quantify the mRNA levels. GAPDH was used as an internal control. Primer sequences are listed in Table 1.

**Table 1:** Primer sequences

Gene	Forward primer (5′–3′)	Reverse primer (5′–3′)
GBP5	CTTAGGCAGTGCTGGGGAGT	GCAAGGGAACAGATGGGATA
NLRP3	AGCCTCAACAAACGCTACAC	CGGGGTCAAACAGCAACT
Caspase-1	ACTGCCCAAGTTTGAAGGAC	GTGGAAGAGCAGAAAGCGAT
GAPDH	CACCCACTCCTCCACCTTTGA	TCTCTCTTCCTCTTGTGCTCTTGC

### ELISA

Plasma caspase-1, IL-1β, and IL-18 levels were measured by enzyme-linked immunosorbent assay kits (ELISA, MultiSciences Biotech Co., Ltd.) according to the manufacturer’s instructions.

### Statistical analysis

Statistical data were analyzed by SPSS 25.0 software ((IBM, Armonk, NY, USA). The data were presented as the mean ± standard deviation. The t-test was used for normally distributed variables. For categorical variables, the chi-square test was employed, and they were presented as n (%). Pearson correlation analysis was employed for determining correlations. Receiver operating characteristic (ROC) curve analysis helped to assess the GBP5’s diagnostic accuracy in IM children with liver injury. The cut-off value was determined using Youden’s index. All values of *p* < 0.05 were considered statistically significant.

## Results

### Transcriptome sequencing of PBMCs in IM patients with liver and non-liver injuries

**Fig. 1:**
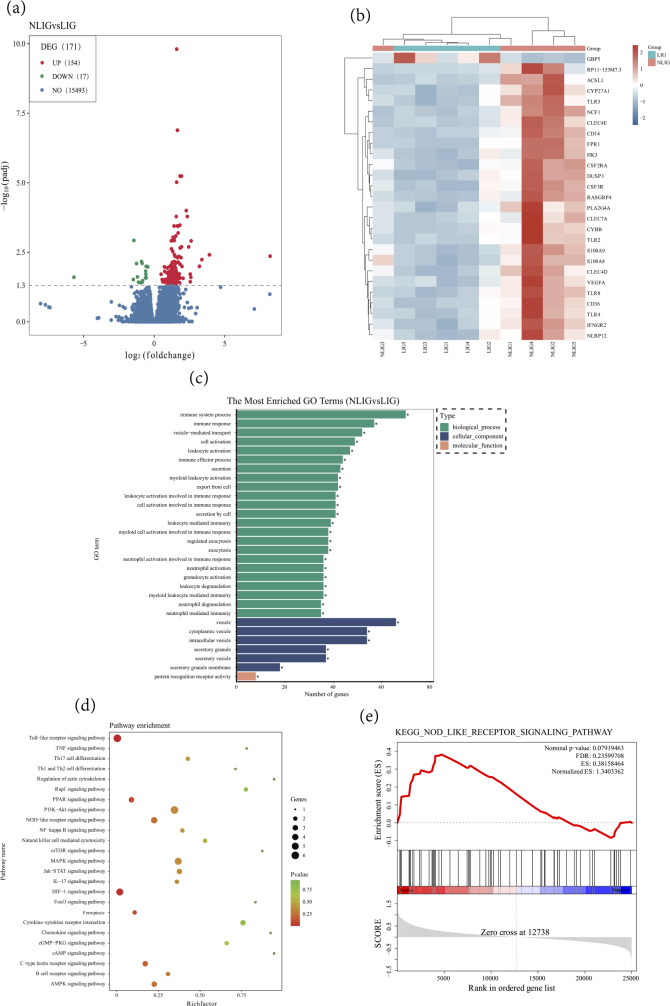
Transcriptome sequencing of PBMCs in IM patients. (**a**) The RNA sequencing results of DEGs in NLIG and LIG were plotted by volcano plot (*n* = 5). (**b**) Clustering heat map of DEGs between NLIG and LIG (*n* = 5). (**c**) GO enrichment analysis showing DEGs of NLIG and LIG. (**d**) Enrichment analysis of signaling pathways of DEGs in the two groups using the KEGG database. (**e**) GSEA correlation plot between IM with liver injury and NOD-like receptor signaling pathway in the RNA sequencing results

Transcriptome sequencing results showed that 171 DEGs were screened in the NLIG as compared to LIG. Among them, 154 DEGs were up-regulated and 17 were down-regulated (Fig. 1a). The clustering heat map showed results of partly significantly different genes, where each column represents a sample and different colors represent the gene expressions in the sample. Low expression was shown in blue; the darker color exhibited reduced expression. The higher expression was denoted by red; the darker color signified enhanced expression (Fig. 1b). Gene ontology (GO) functional enrichment analysis showed that the biological process was highly abundant in the first 30 enrichment items; the most significant of them were the immune system process and immune response (Fig. 1c). Subsequently, 25 pathways were significantly selected for the Kyoto Encyclopedia of Genes and Genomes (KEGG) enrichment analysis. Consequently, the nucleotide oligomerization domain (NOD)-like receptor signaling pathway was the one that was significantly activated(Fig. 1d). Moreover, the NOD-like receptor signaling pathway gene upregulation in LIG was also confirmed by Gene set enrichment analysis (GSEA, Fig. 1e).

### Patient characteristics

No significant differences were seen in mean age, gender, the number of feverish children, duration of fever, and hospital stays between IM children with liver injury and non-liver injury (Table 2). Compared with NLIG, WBC, and L counts, CD3^+^CD8^+^T cells (%), ALT, AST, and LDH were significantly higher in IM children with liver injury. CD3^+^CD4^+^ T cells (%) were lower in the LIG. Other laboratory tests displayed no significant difference (Table 2).

**Table 2:** Clinical and laboratory characteristics of IM children with liver and non-liver injuries

Variable	NLIG(*n* = 30)	LIG(*n* = 30)	t or χ2 value	*p* value
Age, years	4.70 ± 2.13	5.70 ± 2.55	−1.66	0.103
Male, n(%)	15(50.0)	12(40.0)	0.61	0.436
Fever (T > 37.3℃), n(%)	27(90.0)	27(90.0)	0.00	1.000
Duration of fever, days	5.43 ± 3.60	6.50 ± 3.87	−1.11	0.273
Hospital stays, days	7.00 ± 2.23	8.23 ± 3.81	−1.53	0.131
WBC,10^9^ /L	14.56 ± 4.69	18.51 ± 6.12	−2.80	0.007
N,10^9^ /L	4.19 ± 2.25	3.74 ± 1.87	0.83	0.410
L,10^9^ /L	8.98 ± 3.56	13.09 ± 4.55	−3.89	< 0.001
M,10^9^ /L	1.31 ± 0.78	1.53 ± 0.94	−0.91	0.368
IgA, g/L	1.65 ± 0.72	1.75 ± 0.71	−0.56	0.580
IgG, g/L	11.30 ± 2.95	11.99 ± 2.64	−0.95	0.344
IgM, g/L	1.64 ± 0.60	1.93 ± 0.57	−1.97	0.054
CD3^+^,%	83.03 ± 6.93	84.97 ± 6.98	−1.08	0.286
CD3^+^CD4^+^,%	18.74 ± 7.20	13.73 ± 5.52	3.02	0.004
CD3^+^CD8^+^,%	51.41 ± 12.00	65.58 ± 7.01	−5.59	< 0.001
CD3^−^CD19^+^,%	5.19 ± 2.28	4.50 ± 4.24	0.78	0.440
CD3^−^CD(16^+^56)^+^,%	10.17 ± 5.38	9.86 ± 4.12	0.25	0.806
LDH, U/L	452.84 ± 81.49	561.35 ± 115.13	−4.21	< 0.001
ALT, U/L	22.11 ± 6.69	224.18 ± 199.12	−5.56	< 0.001
AST, U/L	38.16 ± 7.60	206.98 ± 155.26	−5.95	< 0.001
TBil,µmol/L	5.59 ± 2.01	6.58 ± 3.37	−1.39	0.172
Plasma EBV-DNA lg(copies/ml)	2.94 ± 1.04	3.10 ± 0.95	−0.63	0.531
Whole blood EBV-DNA lg(copies/ml)	5.25 ± 1.07	5.46 ± 0.94	−0.81	0.421

The data were presented as mean ± standard deviation and n (%). The univariate analyses were performed using a t-test for normally distributed and a chi-square test for categorical variables, respectively. Temperature (T), WBC, N, L, M, IgA, IgG, IgM, LDH, ALT, AST, TBil, plasma EBV-DNA and whole blood EBV-DNA were the study variables

### GBP5 expression was abnormally elevated in IM patients with liver injury

**Fig. 2:**
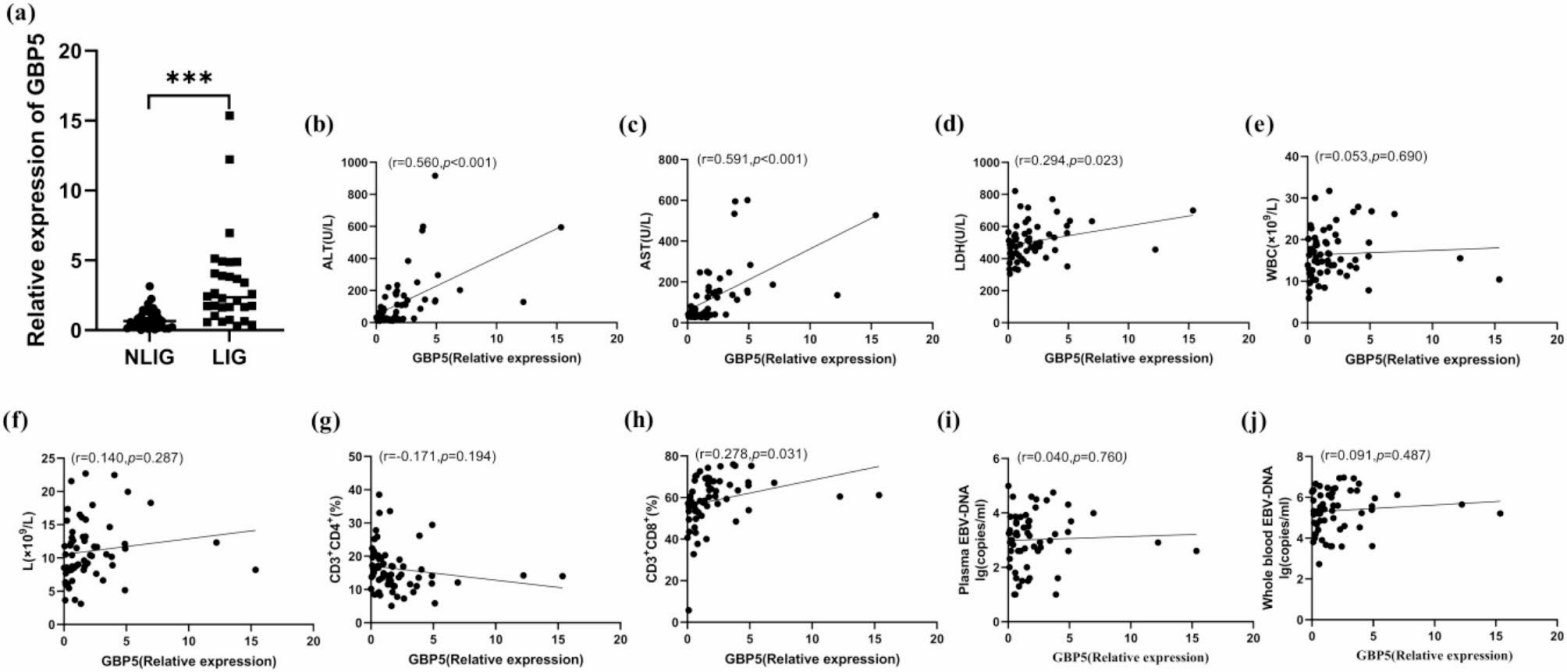
GBP5 was associated with liver injury in IM children. (**a**) The relative GBP5 expression in PBMCs in NLIG and LIG ***p < 0.001. (**b**-**j**) Correlation between the expressions of GBP5 and ALT, AST, LDH, WBC, L, CD3^+^CD4^+^T cells (%), CD3^+^CD8^+^T cells (%), Plasma EBV-DNA lg(copies/ml) and Whole blood EBV-DNA lg(copies/ml) ALT, alanine aminotransferase; AST, aspartate transaminase; LDH, lactate dehydrogenase; WBC, white blood cells, L lymphocyte; PBMCs, peripheral blood mononuclear cells

In comparison with the IM children with NLIG, the relative GBP5 expression in PBMCs was significantly higher in the IM children with LIG [(0.80 ± 0.74) vs. (3.32 ± 3.33), (Fig. 2a)]. Correlation analysis in IM patients showed an association between the GBP5 expressions in PBMCs with serum ALT, AST, LDH and CD3^+^CD8^+^T cells (%). Moreover, the GBP5 level was positively correlated with ALT, AST, LDH and CD3^+^CD8^+^T cells (%, Fig. 2b-j).

### Diagnostic values of GBP5 in IM children with liver injury

**Fig. 3:**
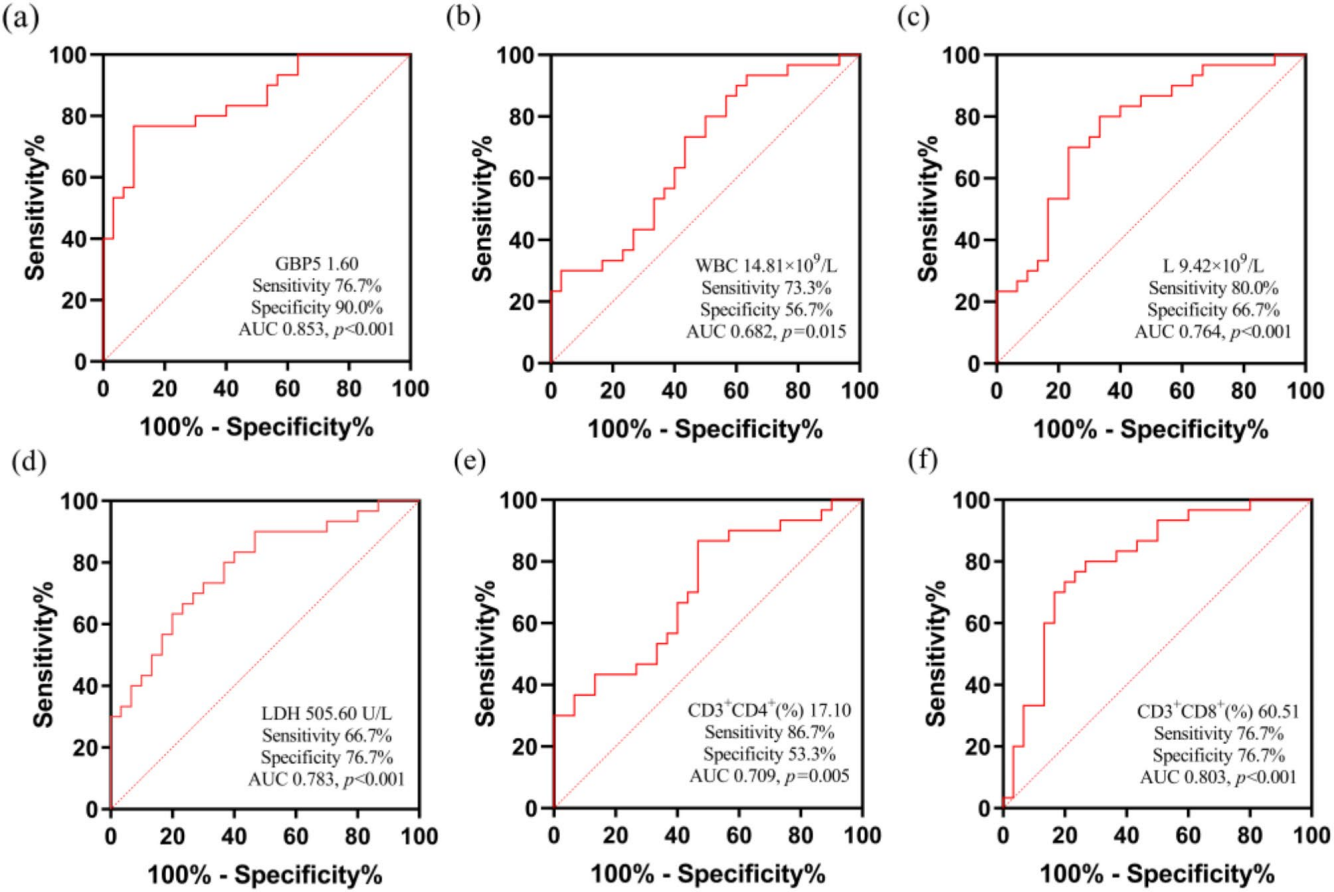
The diagnostic values of GBP5, WBC, L, LDH, CD3^+^CD4^+^T cells (%) and CD3^+^CD8^+^T cells (%) in IM children with liver injury (a-f). WBC, white blood cells; L, lymphocyte; LDH, lactate dehydrogenase

The relative GBP5 expression in PBMCs displayed higher diagnostic accuracy for differentiating IM with liver injury. The GBP5 cut-off value was 1.60, the sensitivity was 76.7%, the specificity was 90.0%, and the area under the curve was 0.853 (*p* < 0.001). This index exhibited better results than WBC, L, LDH, CD3^+^CD4^+^T cells (%), and CD3^+^CD8^+^T cells (%) in determining diagnostic values of IM children with liver injury (Fig. 3a-f).

### NLRP3 inflammasome activation by GBP5 expression in IM children with liver injury

**Fig. 4:**
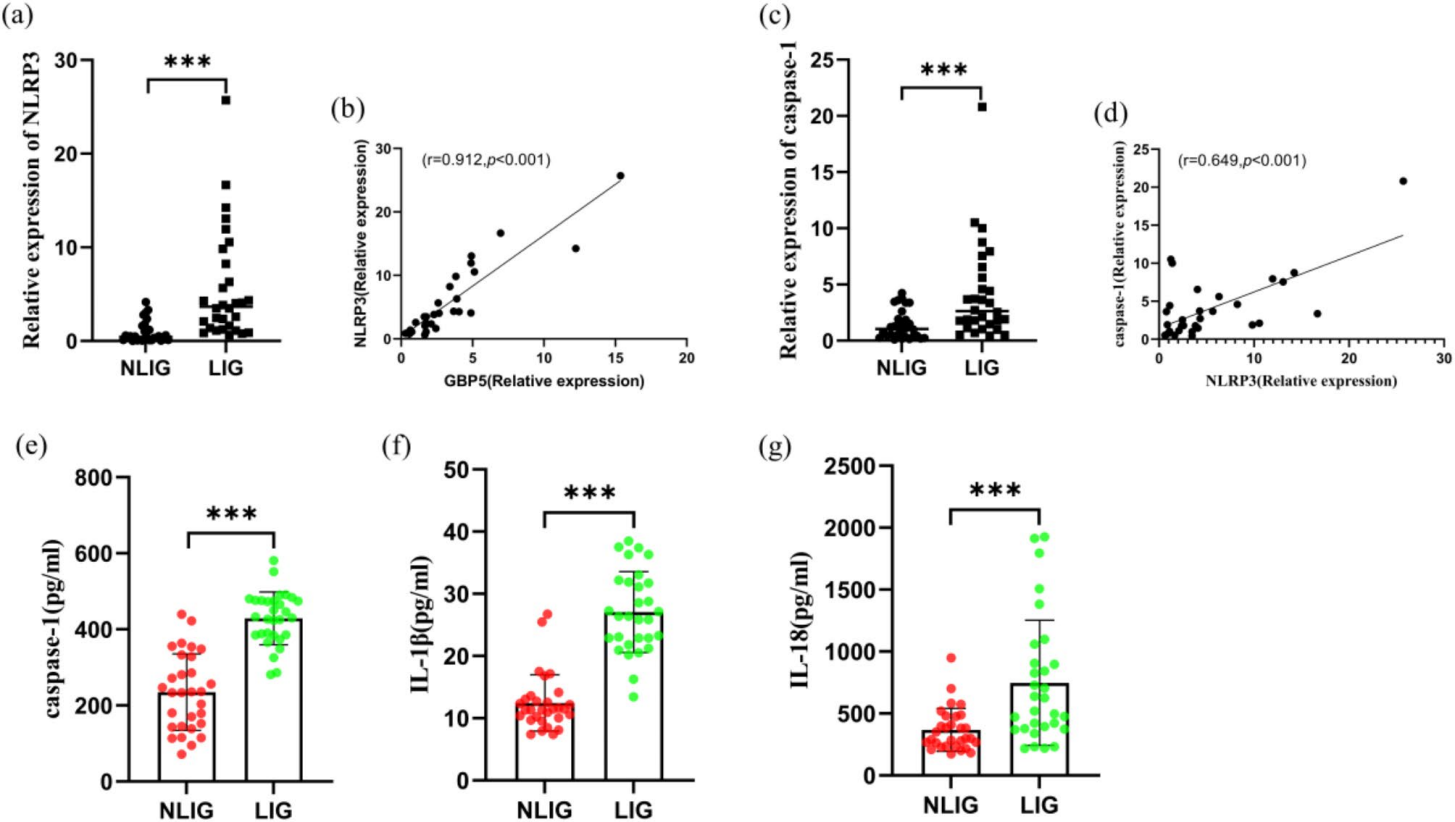
GBP5 aggravated IM-associated liver injury via NLRP3 inflammasome activation. (**a**) The relative NLRP3 expressions in PBMCs in NLIG and LIG ***p < 0.001. (**b**) Correlation between the expressions of GBP5 and NLRP3. (**c**) The caspase-1 expressions in PBMCs in NLIG and LIG ***p < 0.001. (**d**) Correlation between the expressions of NLRP3 and caspase-1 expressions. (**e**-**g**) Plasma levels of caspase-1, IL-1β, and IL-18 were measured by ELISA ***p < 0.001

To explore the underlying mechanism of GBP5 in IM children with liver injury, NOD-like receptor protein 3 (NLRP3) inflammasome was focused. Compared to NLIG, the relative NLRP3 expression in PBMCs was significantly higher in the LIG [(0.93 ± 1.14) vs. (5.63 ± 5.85), Fig. 4a)]. Moreover, the GBP5 and NLRP3 expressions were positively correlated (Fig. 4b). Additionally, the caspase-1 expression in PBMCs was significantly higher in the LIG than NLIG [(4.15 ± 4.28) vs. (1.51 ± 1.46) (Fig. 4c)] while NLRP3 and caspase-1 expressions were positively correlated (Fig. 4d). When compared with NLIG, the plasma levels of caspase-1 [(234.95 ± 100.59pg/mL) vs. (428.95 ± 69.40pg/mL)], IL-1β [(12.45 ± 4.53pg/mL) vs. (27.05 ± 6.50 pg/mL)], and IL-18 [(367.16 ± 173.03pg/mL) vs. (747.27 ± 505.59pg/mL)] were significantly higher in the LIG (Fig. 4e-g). Therefore, these results indicated that GBP5 might significantly regulate IM-associated liver injury through NLRP3 inflammasome activation.

## Discussion

Approximately 50% of pediatric patients with primary EBV infection in childhood exhibit IM. Being a self-limiting lymphoproliferative disorder with a good prognosis, its mortality rate is only 1 ~ 2% [19]. Liver injury is the most common complication in IM pediatric patients. The mechanism of EBV infection-induced liver injury is complicated. At present, the pathological changes in the liver infected by EBV are diffuse lymphocyte infiltration in hepatic sinusoids. EBV does not directly infiltrate hepatocytes, biliary epithelial cells, and vascular endothelial cells. The liver injury might be caused by lymphocyte infiltration and inflammatory factor accumulation post-EBV infection; however, the immune inflammatory response plays an important role in the pathogensis [20]. Primarily involved in immune responses, PBMCs play an important role in regulating host defense against infections [13]. Thus, PBMCs were extracted from IM patients with and without liver injuries in our study to explore the mechanism of liver injury in pediatric IM patients. The transcriptome sequencing analysis showed that 171 DEGs existed between the two groups (Fig. 1a). Additionally, GO functional enrichment analysis indicated that the two DEG groups were mainly enriched in immune system processes and immune responses (Fig. 1c). Innate immunity is the first line of defense against microbial invasion. GBP5, as an effector molecule activated by pathogen infection, plays an important biological role in generating immune response [21, 22]. In this study, Quantitative real-time PCR detected that GBP5 displayed significantly differential expression between the two groups, and was highly expressed in IM patients with liver injury (Fig. 2a). And the expressions of GBP5 were positively correlated with ALT, AST, and LDH (Fig. 2b-d). Therefore, we suggest that GBP5 is significantly involved in the regulation of IM-associated liver injury.

KEGG enrichment analysis showed that the two DEG groups were enriched in the NOD-like receptor signaling pathway (Fig. 1d); the upregulation of the NOD-like receptor signaling pathway gene in LIG was also confirmed by GSEA (Fig. 1e). These results suggest that IM-associated liver injury is related to the immune inflammatory responses and NOD-like receptor signaling pathway. Thus, we suggest that GBP5 can regulate IM-associated liver injury through an NOD-like receptor signaling pathway. Inflammasome is a protein complex, which exists in the cytoplasm of cells. The NOD-like receptor family is an inflammasome and includes the pyrin domain (PYD) of NLRP3. When activated by infections or cellular stress, it forms a complex with apoptosis-associated speck-like protein (ASC) containing a caspase recruitment domain. Consequently, it triggers the cleavage of the pro-caspase-1 into caspase-1, activates caspase-1 and pro-inflammatory cytokine release, and induces inflammatory immune responses [23, 24]. However, excessive or aberrant inflammasome activation can lead to inflammatory diseases. A previous study showed that inflammasome is involved in the pathogenesis of several liver diseases, like non-alcoholic fatty liver disease, hepatitis B, hepatic fibrosis, and liver cancer [25]; it is also closely related to acute liver injury caused by various etiologies [26]. In this study, the NLRP3 and caspase-1 expressions in PBMCs were significantly higher in the LIG than in NLIG (Fig. 4a, c). Moreover, in comparison with the NLIG, the plasma levels of caspase-1, IL-1β, and IL-18 were significantly enhanced in the LIG (Fig. 4e-g). Therefore, we suggest that NLRP3 inflammasome activation promotes liver injury in IM pediatric patients by releasing inflammatory factors, like IL-1β and IL-18.

GBP5 is crucial for the assembly and activation of inflammasomes and is an upstream regulator that mediates NLRP3 inflammasome activation [27, 28]. The N-terminal GTPase domain of GBP5 protein binds to the caspase activation and recruitment domain (CARD) of NLRP3 inflammasome, thereby promoting the assembly of NLRP3 inflammasome and activating signaling pathways downstream [29]. We observed that the GBP5 and NLRP3 expressions were positively correlated, while NLRP3 and caspase-1 expressions were significantly associated (Fig. 4b, d). Thus, we propose that GBP5 might regulate IM-associated liver injury through NLRP3 inflammasome activation. However, other pathways might also play a significant role. Recent studies have found that the NF-κB signaling pathway and AIM2 inflammasome are involved in the occurrence of liver injury caused by various etiologies [30, 31]. Therefore, GBP5 may also regulate IM-associated liver injury by activating the NF-κB signaling pathway or AIM2 inflammasome simultaneously. These conjectures will be further verified in our follow-up research. Besides IM-associated liver injury, the mechanism of GBP5 in activating the NLRP3 inflammasome plays a key role in the pathogenesis of inflammatory bowel diseases and arthritis [32, 33].

The IM-induced liver injury is not the direct impairment of EBV to hepatocytes. EBV infection enhances the lymphocyte population. The increased CD8^+^T cells are captured by the liver, which produces cytokines and inflammatory mediators that lead to indirect liver injury [34, 35]. However, the precise mechanism is still unknown. This study found that compared with the NLIG group, the proportion of CD3^+^CD8^+^T cells in the LIG was increased, and the proportion of CD3^+^CD4^+^T cells was decreased. These results were consistent with the results of previous studies [36, 37]. GBP family members are widely found in several immune cells, and GBP5 is expressed in monocytes, macrophages, T and B lymphocytes, as well as NK cells [38, 39]. This study found that GBP5 expression was positively correlated with the CD3^+^CD8^+^T cells (Fig. 2h). Therefore, we consider that CD8^+^T cells proliferate and infiltrate the liver post-EBV infection. Enhanced GBP5 expression in CD8^+^T cells promotes the assembly and activation of NLRP3 inflammasome, thereby leading to caspase-1 activation, release of IL-1β and IL-18, and a resultant liver injury.

Although the incidence of IM has been increasing in recent years [40], the methods of predicting and evaluating liver injury in IM children are still limited. Therefore, it is necessary to find a specific predictor for determining the risk of liver injury in IM children. Our results showed that the GBP5 expression in PBMCs has a high diagnostic accuracy for differentiating IM with liver injury and is better than other indicators (Fig. 3). GBP5 is highly expressed in IM-associated liver injury, and the expressions of GBP5 are positively correlated with ALT and AST. GBP5 has shown higher specificity in detecting liver injury related to EBV induced hepatitis in children, it is considered to be the cause of liver injury in children with IM. While traditional markers such as ALT, AST, and LDH can be elevated in IM-associated liver injury, the expression level of GBP5 may change at an earlier stage of the disease process. It can evaluate whether IM children have liver injury at an earlier stage. Therefore, GBP5 can be a specific predictor that helps identify whether IM children have liver injury or not, and it may become a biomarker for evaluating the prognosis of liver injury in the future. However, this study had a few limitations. Firstly, we revealed that GBP5 was highly expressed in IM children with liver injury. Although GBP5 was significantly expressed in CD8^+^T cells, we did not isolate CD8^+^T cells and verify GBP5 expression in those cells. Since we did not obtain liver tissues from IM patients, we could not determine any difference in GBP5 expression in hepatocytes between NLIG and LIG. In future studies, we will initially select 20 children with IM and divide them into two groups (10 cases in each group) according to the appearance of liver injury. No significant difference was observed in sex and age in each group. Liver tissue specimens will be obtained by liver biopsy. Quantitative real-time PCR and immunohistochemistry will be used to detect the expression of GBP5, NLRP3, and caspase-1 in liver tissues. Hematoxylin and eosin staining will be used to observe the infiltration of inflammatory cells and the liver cells damage of the liver tissues of two groups. Secondly, this study was a clinical research study with a small sample size, so the sample size should be increased in the future to support our results’ reliability. Since our cell experiment did not confirm the physiology of GBP5 regulating IM-associated liver injury through NLRP3 inflammasome activation, the required cell research will be finished in additional studies.

## Conclusion

Our findings demonstrated that GBP5 expression was increased in IM patients with liver injury. Additionally, GBP5 displayed enhanced diagnostic accuracy in differentiating IM in children with and without liver injuries. Thus, we suggest that CD8^+^T cells proliferate and infiltrate the liver after EBV infection. However, the enhanced GBP5 expression in CD8^+^T cells can induce liver injury, which might be dependent on the NLRP3 inflammasome activation. Hence, our study provided novel insights into the pathogenesis of IM pediatric patients with liver injury.

## Data Availability

All analyzed datasets are available from the corresponding author upon request.

## Abbreviations

EBV: Epstein-Barr virus
IM: Infectious mononucleosis
NLIG: Non-liver injury group
LIG: Liver injury group
GBP5: Guanylate-binding protein 5
PBMCs: Peripheral blood mononuclear cells
DEGs: Differential genes
GO: Gene ontology
KEGG: Kyoto Encyclopedia of Genes and Genomes
GSEA: Gene set enrichment analysis
NLRP3: NOD-like receptor protein 3
WBC: White blood cell
N: Neutrophil
L: Lymphocyte
M: Monocyte
IgA: Immunoglobulin A
IgG: Immunoglobulin G
IgM: Immunoglobulin M
LDH: Lactate dehydrogenase
ALT: Alanine aminotransferase
AST: Aspartate transaminase
TBil: Total bilirubin
T: Temperature
